# Maternal Variables as Determinant of Fetal Growth: Study Protocol on Customized Fetal Growth Charts in Malaysia (GROW-My)

**DOI:** 10.3389/fmed.2021.592462

**Published:** 2021-05-25

**Authors:** Muhammad Za'im Sahul Hameed, Rosnah Sutan, Zaleha Abdullah Mahdy, Azmi Mohd Tamil, Saperi Sulong

**Affiliations:** ^1^Department of Community Health, Faculty of Medicine, The National University of Malaysia, Kuala Lumpur, Malaysia; ^2^Department of Obstetrics and Gynaecology, Faculty of Medicine, The National University of Malaysia, Kuala Lumpur, Malaysia

**Keywords:** small for gestational age, low birth weight, growth chart, antenatal monitoring, fundal height, symphysiofundal height

## Abstract

Adverse perinatal outcomes such as stillbirth, low birth weight and small for gestational age are still reported to be of high prevalence despite advanced healthcare technology and good quality hospital services in Malaysia. The purpose of this study is to create a model to evaluate individualized birth weight customized for maternal characteristics in a Malaysian population. Three phases are involved in designing the customized fetal growth chart (GROW-My). Baseline data is collected from previous pregnancies in the UKM Medical Centre from year 2010 to 2017. Specific maternal attributes were screened for its completeness and validity, namely maternal height and weight at booking, maternal ethnicity and parity, and the baby's birth weight, for all singleton pregnancies. The design and construction of a Malaysian customized fetal growth chart, Growth Related Optimal Weight (GROW-MY) was based on baseline birth data. The customized chart is used in the implementation phase for testing its feasibility, taking into consideration feedback from caregivers and patients before and after implementation. The current study provides staunch information and data regarding the needs and strategies for using maternal variables for estimating birth weight and the risk of being small for gestational age, in order to facilitate screening and appropriate management. With improved diagnosis of fetal growth restriction, medical care and treatment costs can be reduced.

## Introduction

Undiagnosed small for gestational age (SGA) is a risk factor for fetal growth restriction (FGR) and stillbirths. Pregnancies with FGR have an 8-fold higher risk of stillbirth (1). Undetected FGR increases the risk of stillbirth even further (2). Currently, the Malaysian health system still uses population-based growth charts, which have been proven to be inaccurate (3). The growth charts used are not customized to individuals. Every individual is assumed to have the same fetal growth and the fact that maternal height, weight, parity, ethnicity, and fetal gender affects fetal growth, is disregarded. Many studies have shown that maternal ethnicity, weight, and height have significant effects on birth weight (4–7). Even in Asian countries such as India, the use of customized growth charts has been shown to be more accurate and precise in diagnosing fetal anomalies (8). It is a promising prospect for a country such as Malaysia where a portion of the population originated from India.

The main cause of SGA is either simply being constitutionally small, or FGR (9). SGA due to FGR is associated with a greater risk of early childhood mortality and morbidities than normal birth weight. The cause of stillbirth in Malaysia is not easy to identify because of limitations in the classification system and post mortem assessment. As an upper middle-income country, Malaysia reported a stillbirth rate in the range of 4–5 per 1,000 births. About 30–40% were recorded as normally formed macerated stillbirths (10). In 2010, an estimated 32.4 million infants are born SGA in low income and middle-income countries, from which 10.6 million were born at term (11). The prevalence of Malaysian low birth weight (LBW) babies was recorded to have reached a plateau since 1990s despite improving socioeconomic status and advances in the healthcare delivery system, with a higher prevalence among Indians (10).

The use of a customized growth chart is expected to improve the accuracy of diagnosing fetal growth anomalies in pregnancy, leading to more efficient delivery of care. No attempt has yet been made to design a customized growth chart using a Malaysian population based data and implementing it using symphysiofundal height (SFH) measurement for monitoring the incidence of SGA and preventing stillbirth. We describe the development of a study protocol to construct a customized fetal growth chart for Malaysia based on maternal parity, height, weight and ethnicity, and fetal gender, and to study its feasibility in clinical practice.

## Methods

Our research protocol is designed in three phases. The first phase was a systematic review of literature on customized fetal growth charts. In the second phase of the study, data regarding mothers who delivered at our hospital within the period from 2010 to 2017, specifically their ethnicity, parity, height and weight, and fetal gender and birth weight, were obtained from the hospital obstetric registry to create a predicted model for optimal fetal growth trajectory for construction of a customized fetal growth chart. This growth chart utilizes maternal variables in calculating fetal growth. For example, mothers with higher weight and height are expected to have heavier babies, and mothers from European ethnicity are likely to have heavier babies than those of Indian ethnicity. This allows more accurate prediction of fetal growth as large mothers are not misdiagnosed with large fetuses and small mothers are not misdiagnosed with SGA. In the third phase of this study, the customized growth charts will be implemented and tested in a hospital setting. The charts will be printed individually for all mothers who are subsequently followed up until delivery. Mothers selected for this study do not have any known underlying medical illness, are at 26 weeks' pregnancy or earlier, with singleton pregnancies. Questionnaires are posed to mothers and clinicians to assess the feasibility of the new growth chart. SGA detection rate is measured and compared for both population based growth chart and the customized chart.

### Research Hypothesis

Two hypotheses are tested in this study. The first is that maternal ethnicity, parity, height and weight, and fetal gender, affect the outcome of pregnancy (Phase 2). The second is that the customized fetal growth chart is feasible for use in a hospital setting and improves perinatal outcome (Phase 3).

### Phase 1: Systematic Review

A systematic review was conducted to evaluate previous studies that used customized fetal growth charts, so as to help understand the benefits of such charts, how to perform a study utilizing them, and their limitations. Articles were searched through PubMed, EMBASE and the Cochrane Library between 2007 and 2020, focusing on interventional and observational studies comparing the adverse outcomes of pregnancy, specifically LBW and SGA neonates, when classified in accordance with customized vs. population-based growth charts. Intrauterine fetal death (IUFD), FGR, and perinatal mortality were the outcomes evaluated.

### Phase 2: Baseline Obstetric Registry Data

Data was obtained from the labor ward ObsCentral (Mediclink Systems [Malaysia] Private Limited) database. Birth data is routinely entered into the database by obstetric medical officers. Verification of completeness of the data is done by hospital record officers. Data of healthy mothers who were Malaysian citizens with normal singleton pregnancies was included into this study. The data was screened for missing variables, extreme values and pathologies. Extreme values such as head circumference beyond 30–50 cm, birth weights beyond 2,500–4,000 g, maternal heights beyond 130–175 cm, maternal weight beyond 30–150 kg, maternal age beyond 15–50 years and gestational ages beyond 175–308 days were all excluded. Pathologies such as diabetes mellitus, syndromes, hypertension, eclampsia, preeclampsia and asthma were excluded.

Data of deliveries from October 2010 to December 2017 were collected. There were 50,371 sets of data. Using STATA, the data was screened for invalid values.

The birth parameters were based on the normal healthy birth weight. Maternal age was limited to childbearing age. Gestational age was selected based on viable fetal age. The acceptable range of parameters of height and weight of women in Malaysia were based on a study on distribution of body weight, height, and body mass index in a national sample of Malaysian adults by Lim TO et al. in 2008 (12). We chose only singleton fetuses as this was the target of the study.

A total of 19,823 (39.35%) variables were invalid and removed, and 30,548 variables remained (Table 1). The database was imported into STATA from Excel spreadsheet. For convenience, gestational period was divided into weeks and days. A total of 20.33% of patients who had diabetes mellitus, syndromes, hypertension, eclampsia, and asthma were removed (Table 2) as a sample of healthy Malaysian mothers was targeted for the study. Using STATA, words and phrases including “SGA,” “smaller than date” and “small for gest” were searched for.

**Table 1:** Invalid values filtered.

**Variable**	**Parameter**	**Frequency (%)**
Birth weight	2,500–3,500 g	387 (0.77)
Maternal height	130–175 cm	1,756 (3.49)
Maternal weight	30–200 kg	5,179 (10.28)
Maternal age	15–49 years	450 (0.89)
Gestational age	175–308 days	132 (0.26)
Number of fetus	Singleton	492 (0.98) Twin/Triplets excluded
Total		8,396 (16.67)

**Table 2:** Pathologies filtered.

**Pathology**	**Frequency (%)**
Diabetes	7,964 (15.81)
Syndromes	142 (0.28)
Hypertension	497 (0.99)
Eclampsia	679 (1.35)
Asthma	959 (1.90)
Total	10,241 (20.33)

**Figure 1:**
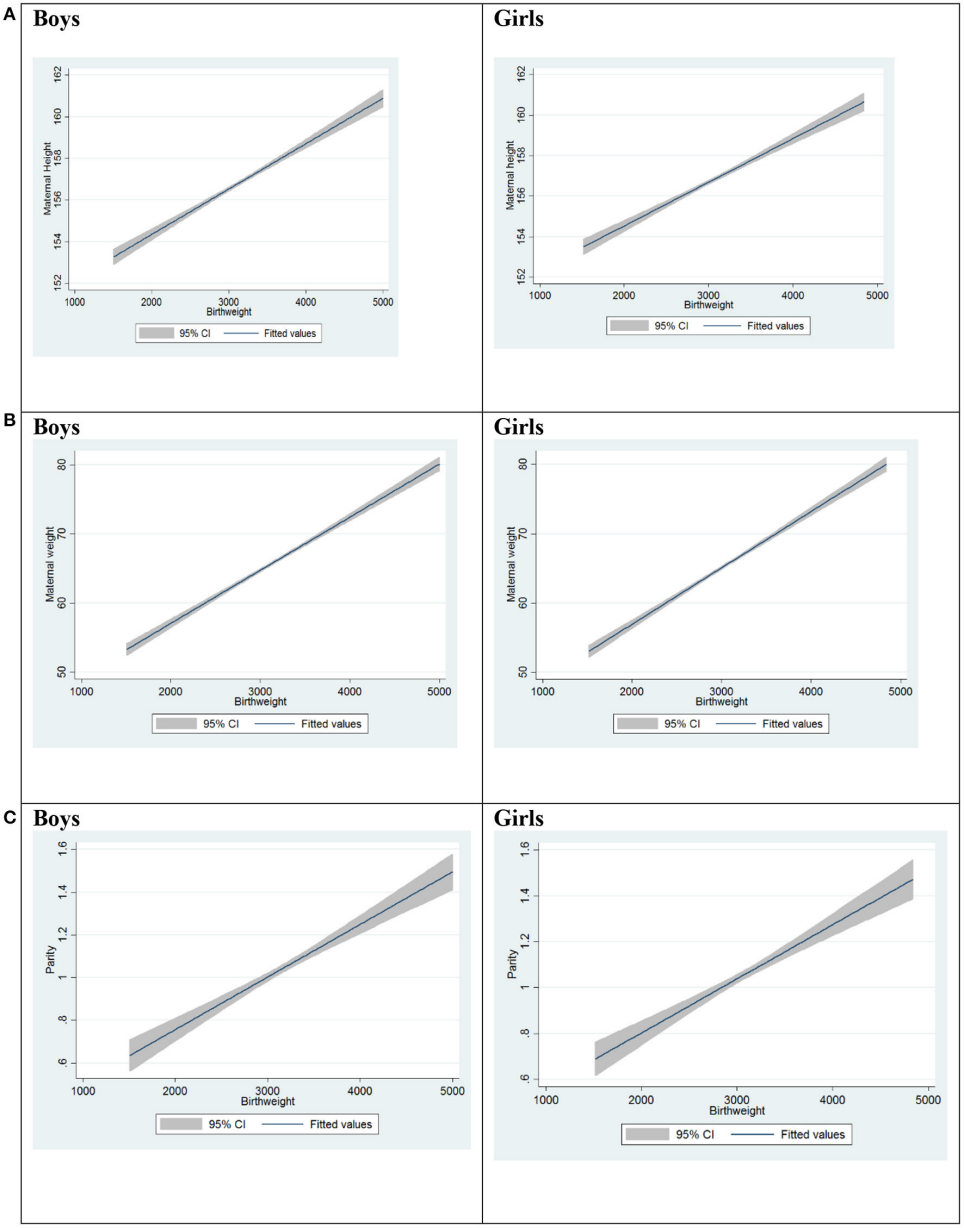
Maternal height vs. birth weight, maternal weight vs. birthweight and parity vs. birth weight, by fetal gender. **(A)** Maternal height vs. birth weight. **(B)** Maternal weight vs. birth weight. **(C)** Parity vs. birth weight.

**Figure 2:**
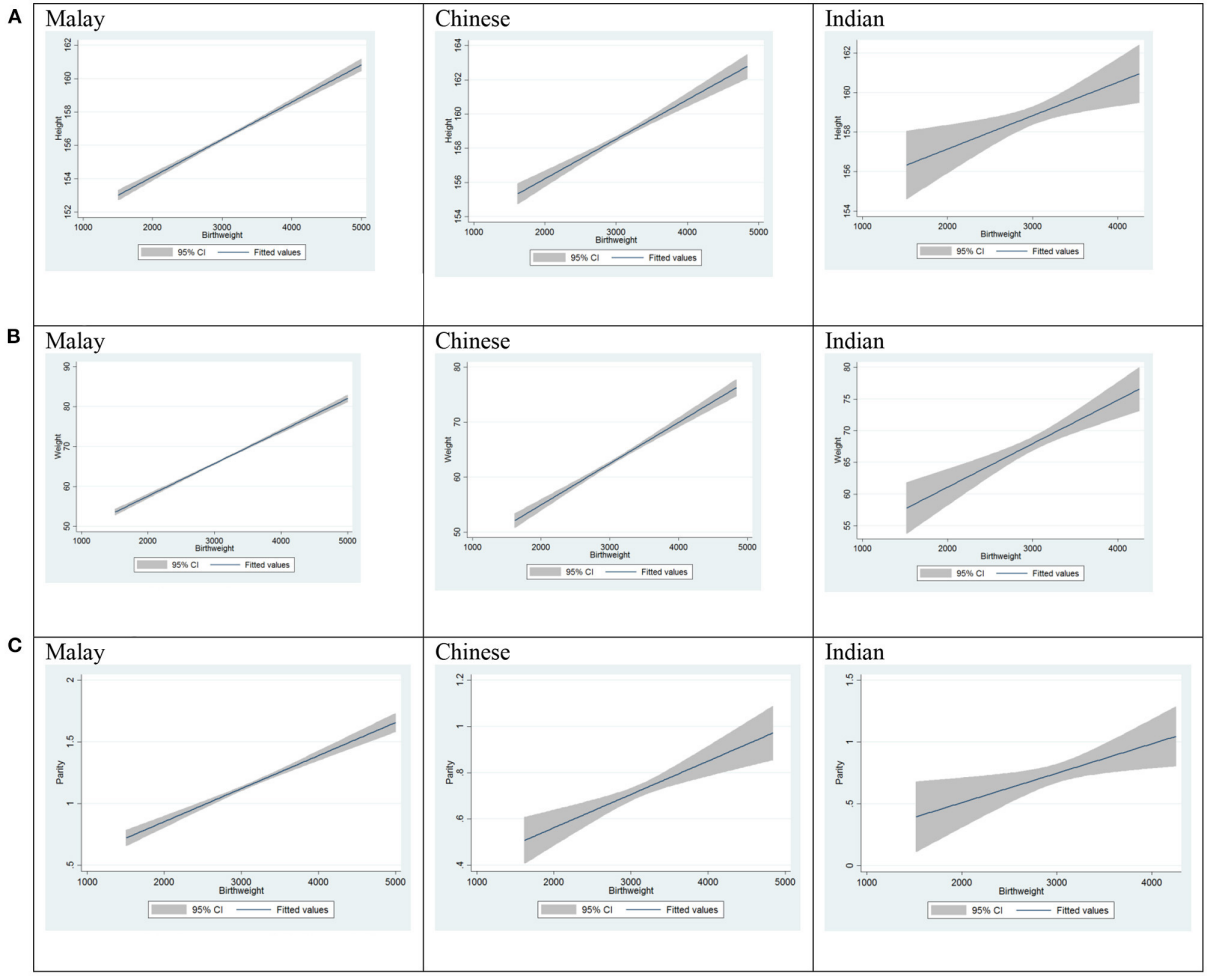
Maternal height vs. birth weight, maternal weight vs. birth weight and parity vs. birth weight, by ethnicity. **(A)** Maternal height vs. birth weight. **(B)** Maternal weight vs. birth weight. **(C)** Parity vs. birth weight.

Relationship between the variables was found using multiple regression of the data collected. The regression analysis assumes that there is a linear relationship between the outcome and independent variables. In this case, the independent variables were maternal height, weight, parity, ethnicity, and fetal gender. The calculations were made in STATA 13. Using stepwise multiple regression, findings are as shown in Table 3. Multiple linear regression analysis showed that there was a linear correlation between maternal height, weight and parity, with birth weight for both fetal genders—male and female. There was also a linear correlation between the same variables when it comes to ethnicities: Malay, Chinese and Indian. Figure 1 shows best fit lines with confidence intervals between maternal height and birth weight, maternal weight and birth weight, and parity and birth weight, by fetal gender. A positive correlation is demonstrated between the two variables. Figure 2 shows best fit lines with confidence intervals by ethnicity. The customized growth chart (GROW-My) is designed based on how maternal height, weight, ethnicity and parity, and fetal gender, affect fetal growth, using the findings from Phase 2 (Table 3).

**Table 3:** Results of multiple linear regression using maternal variables against birth weight.

	**Coefficient**	**Standard error**	***P*-value**	**[95% Confidence interval]**	
				**Minimum**	**Maximum**
Parity	108.99	7.15	0.00	94.97	123.01
Weight	5.81	0.19	0.00	5.43	6.18
Height	6.66	0.39	0.00	5.90	7.43
Fetal gender	92.93	4.13	0.00	84.83	101.03
Chinese	−3.54	5.07	0.48	−13.48	6.39
Indian	−74.12	12.38	0.00	−98.39	−49.86

*Note: Malay ethnicity, median weight 64 kg, median height 163 cm, parity 0 and gender neutral were taken as the reference points*.

From the data of 30,548 patients, we found that only 357 (1.17%) babies were diagnosed as SGA as documented in the ObsCentral database. On the other hand, when the patients were screened based on the customized fetal growth centile that we created, a larger number of 1,267 (4.15%) babies were found to have SGA.

#### Baseline SGA Detection Rate

In a preliminary analysis of retrospective birth data from UKMMC, out of 3,855 singleton pregnancies in 2018, 199 (2.5%) were diagnosed by clinicians as SGA. When 401 randomly selected cases that were not diagnosed with SGA by clinicians were tested using GROW-My, we found that 136 (34%) were missed SGA cases. Out of 199 cases considered to be SGA at birth, 185 were SGA by GROW-My standards. Hence, odds ratio analysis revealed that the customized fetal growth chart has a sensitivity of 0.93 and a specificity of 0.66. The positive predictive value was therefore 0.58 while the negative predictive value was 0.95.

### Phase 3: Feasibility Study of the Customized Fetal Growth Chart

Feasibility testing of the customized fetal growth chart will be carried out in a hospital setting.

#### Study Environment and Population

This study will be conducted at the UKM Medical Centre (UKMMC), a hospital setting. Women with singleton pregnancies without pathologies at 26 weeks' gestation or earlier will be enrolled into the study.

#### Sample Size Calculation

Sample size was calculated based on outcome study and the observation of SGA as a risk factor for stillbirth. From a study by Hari Krishnan et al. (13), using the rate of stillbirth among SGA babies:

*p*1 = Proportion of sample from no risk (non-SGA) population with macerated stillbirth (MSB) before term = 2.26%; and
*p*2 = Proportion of sample from at risk (SGA) population with MSB before term = 16.41%
Hence, relative risk = 7.

Using Epi Info^TM^ (Centers for Disease Control and Prevention, Atlanta, Georgia, USA) calculator (Epitools) (14), the required sample size is 154.

Estimating a dropout rate of 30%, the minimum sample size required is 200 cases.

#### Inclusion Criteria

Malaysian citizens with normal singleton pregnancies who are registered for antenatal follow-up and delivery in UKMMC, will be invited to participate in the study. Recruitment starts during the second trimester and the women will be followed up until after delivery.

#### Exclusion Criteria

Any pregnancy with fetal structural or chromosomal anomalies or preexisting maternal disease such as hypertension, diabetes mellitus, renal disease or autoimmune disorders, or development of obstetric complications such as preeclampsia and gestational diabetes mellitus, are excluded.

#### Data Collection Method

The new customized fetal growth chart (GROW-My) will be implemented in the hospital. It will be printed and kept in the mothers' antenatal records for fetal growth charting. The instrument for collecting data on feasibility of the growth chart is a researcher-developed questionnaire regarding the mothers' and clinicians' attitudes toward using GROW-My.

## Discussion

The use of customized fetal growth charts based on maternal characteristics is increasing in developed countries such as the UK, USA, Canada and New Zealand, suggesting that they are becoming the standard practice. They allow for more accurate diagnosis of pathological events in the growing fetus (4). However, there has been no attempt so far to customize fetal growth charts according to maternal characteristics among the ethnic groups in the Far East or South East Asian region. We believe our study will be the first to do so in this region.

Malaysia is a multiethnic nation with over 60% Malay majority (15). A customized fetal growth chart is currently non-existent for the Malay ethnicity. Our study is the first to include the Malays, together with other ethnic groups in Malaysia—Chinese, Indian and others, which make up 22.6, 6.8, and 1.0% of the population respectively. The Malay ethnicity in our customized fetal growth chart may be applicable on a wider scale within the Malay Archipelago, which includes Indonesia with a huge Malay population, Singapore and Brunei.

The concept of a universal standard such as Intergrowth-21 has been challenged from the perspective of developmental origins and fetal adaptive responses as many biologic and cultural factors can influence fetal growth that should not be viewed as abnormal (16). In a multinational study involving 1.2 million term pregnancies by Francis et al. (17), it was confirmed that the significant differences in mean birth weights and SGA rates between ten country cohorts using the Intergrowth-21 birth weight standard was not due to pathological factors as represented by stillbirth rates. The different SGA rates simply reflected variation in maternal characteristics.

Our study did not consider paternal characteristics as determinants of fetal growth. The influence of paternal characteristics appears to be a subject of controversy. In a study by Ghi et al. (18), it was found that paternal height significantly affected fetal growth. On the contrary, a recent study by Skåren et al. (19) revealed that it is maternal and not paternal factors that determine fetal growth in both genders. In the Intergrowth-21 study, fathers of infants born LGA were reported to be taller and heavier but it was the maternal BMI that had a dominant influence on LGA (20).

Whilst studies in developing countries such as India demonstrated that customized fetal growth charts yield a higher SGA detection rate compared to population based charts (8), at the other end of the spectrum, usage of customized curves allows differentiation between constitutional LGA and true fetal overgrowth, correcting the overall proportion of LGA babies (21).

The feasibility and statistical benefit of customizing ultrasound growth charts have been demonstrated in another study (22).

Our study will be conducted in a single urban center, which is a tertiary teaching hospital. This hospital receives not only patients living within our catchment area but also referrals from elsewhere in Malaysia. Malaysia has an urban to rural ratio of 4 in 1 for with regard to population accessibility to health facilities within 5–10 km (23). In addition, there is no significant difference in maternal nutritional status between urban and rural populations that is anticipated to affect generalization of our study findings (24).

The current study provides strong initial information and data regarding the need for assessing the feasibility of implementing a customized fetal growth chart system in Malaysian hospitals in order to better monitor pregnancy and subsequently improve perinatal outcome. We have used a large data set of births in the UKMMC over a period of 8 years to produce birth-weight-for-gestational-age centile charts. These centiles from births occurring between 2010 and 2017 provide a more valid tool with which to assess fetal growth. We hope the output of our research will help medical and health care providers diagnose SGA more accurately, thus improving perinatal outcome.

## Declarations

We hereby confirm that this assignment is our own work, is not copied from any other person's work (published or unpublished), and has not previously been submitted for assessment either at Frontiers or elsewhere.

## Ethics Statement

This study has been approved by the Research Ethics Committee of the National University of Malaysia.

## Author Contributions

MZ and RS constructed the idea for writing the manuscript and the study protocol. MZ conducted the literature review to compliment the manuscript and as planning for data synthesis. RS verified and edited the flow of the manuscript and study protocol. ZM, AT, and SS proof read the manuscript. All authors contributed to the article and approved the submitted version.

## Conflict of Interest

The authors declare that the research was conducted in the absence of any commercial or financial relationships that could be construed as a potential conflict of interest.
